# Bilateral testicular infiltration as the initial presentation of NRAS-mutant juvenile myelomonocytic leukemia: rapid molecular remission following azacitidine and haploidentical stem cell transplantation

**DOI:** 10.1016/j.lrr.2026.100601

**Published:** 2026-07-23

**Authors:** Yousef VatanParast

**Affiliations:** Shafa Hospital, Department of Genetics, Ahvaz, Iran

**Keywords:** Juvenile myelomonocytic leukemia, NRAS mutation, Testicular infiltration, Extramedullary leukemia, Azacitidine, Hematopoietic stem cell transplantation

## Abstract

**Background:**

Juvenile myelomonocytic leukemia (JMML) rarely manifests with extramedullary involvement beyond spleen, liver, or skin; testicular infiltration at diagnosis is unreported. We describe a Novel Case of bilateral testicular leukemic infiltration as the initial presentation of NRAS-mutant JMML in a toddler, with rapid remission following azacitidine bridging and haploidentical HSCT.

**Case presentation:**

A 2-year-old boy presented with pallor, abdominal distension, and bilateral scrotal swelling. Labs: leukocytosis (58 × 10⁹/L, monocytes 12.8 × 10⁹/L), anemia (Hb 8.2 g/dL), HbF 22%. BM confirmed JMML with NRAS p.G12D (VAF 42%). No pathogenic variants were detected in KRAS, PTPN11, CBL, or NF1. Conventional cytogenetic analysis demonstrated a normal male karyotype (46,XY). US: testes enlarged (18.8/18.2 mL) with hypoechoic infiltration. Azacitidine (75 mg/m²/d × 5, 2 cycles) reduced counts and size. Paternal haplo-HSCT (Flu-Treo-TT conditioning, PTCy) engrafted D + 18; testes normal at 6 weeks, molecular remission (NRAS-) at 3 months.

**Conclusions:**

This novel case underscores the efficacy of azacitidine and haploidentical HSCT for NRAS-mutant JMML with testicular involvement. Routine genital examination and ultrasound are recommended for male patients.

**Abbreviations**
JMML: Juvenile myelomonocytic leukemia
HSCT: Hematopoietic stem cell transplantation
PTCy: Post-transplant cyclophosphamide
VAF: Variant allele frequency
ddPCR: Droplet digital polymerase chain reaction
GVHD: Graft-versus-host disease
STR-PCR: Short tandem repeat polymerase chain reaction

## 1 Introduction

Juvenile myelomonocytic leukemia (JMML) is a rare, aggressive myelodysplastic/myeloproliferative neoplasm of early childhood characterized by constitutive RAS/MAPK pathway activation [1]. Classic extramedullary sites include spleen, liver, and skin [2], but gonadal involvement at diagnosis has never been documented. Exhaustive PubMed and Scopus searches using the terms “juvenile myelomonocytic leukemia” OR “JMML” AND “testicular” OR “testis” OR “gonadal” returned no prior cases, confirming a genuine gap in the phenotypic spectrum of this myeloid disorder.

The patient met all diagnostic criteria of the 2022 WHO classification [3]. Somatic mutations in the RAS pathway are detected in >95% of cases [4], and NRAS mutations (15–25% of patients) are associated with more aggressive behavior and inferior survival after HSCT [5]. Testicular infiltration is a recognized sanctuary site in acute lymphoblastic leukemia [6], but its occurrence in myeloid malignancies at diagnosis is exceptional. This case expands the known extramedullary tropism of JMML and suggests that RAS-pathway hyperactivation may enable leukemic homing to immune-privileged niches, possibly via CXCR4/CXCL12 signaling [7].

Allogeneic HSCT remains the only curative therapy [8]. Haploidentical HSCT with PTCy is a safe and effective alternative when an HLA-matched donor is unavailable [9], and azacitidine is increasingly used as bridging therapy to reduce disease burden before transplant [10].

## 2 Case report

A previously healthy 2-year-old boy presented with a three-week history of pallor, abdominal distension, and painless bilateral scrotal swelling. Examination revealed hepatomegaly (5 cm below costal margin), splenomegaly (6 cm), and firm, non-tender testes measuring approximately 4 × 3 cm each. No swollen nodes, no telltale rashes, no NF1 stigmata like café-au-lait patches.

**Fig. 1:**
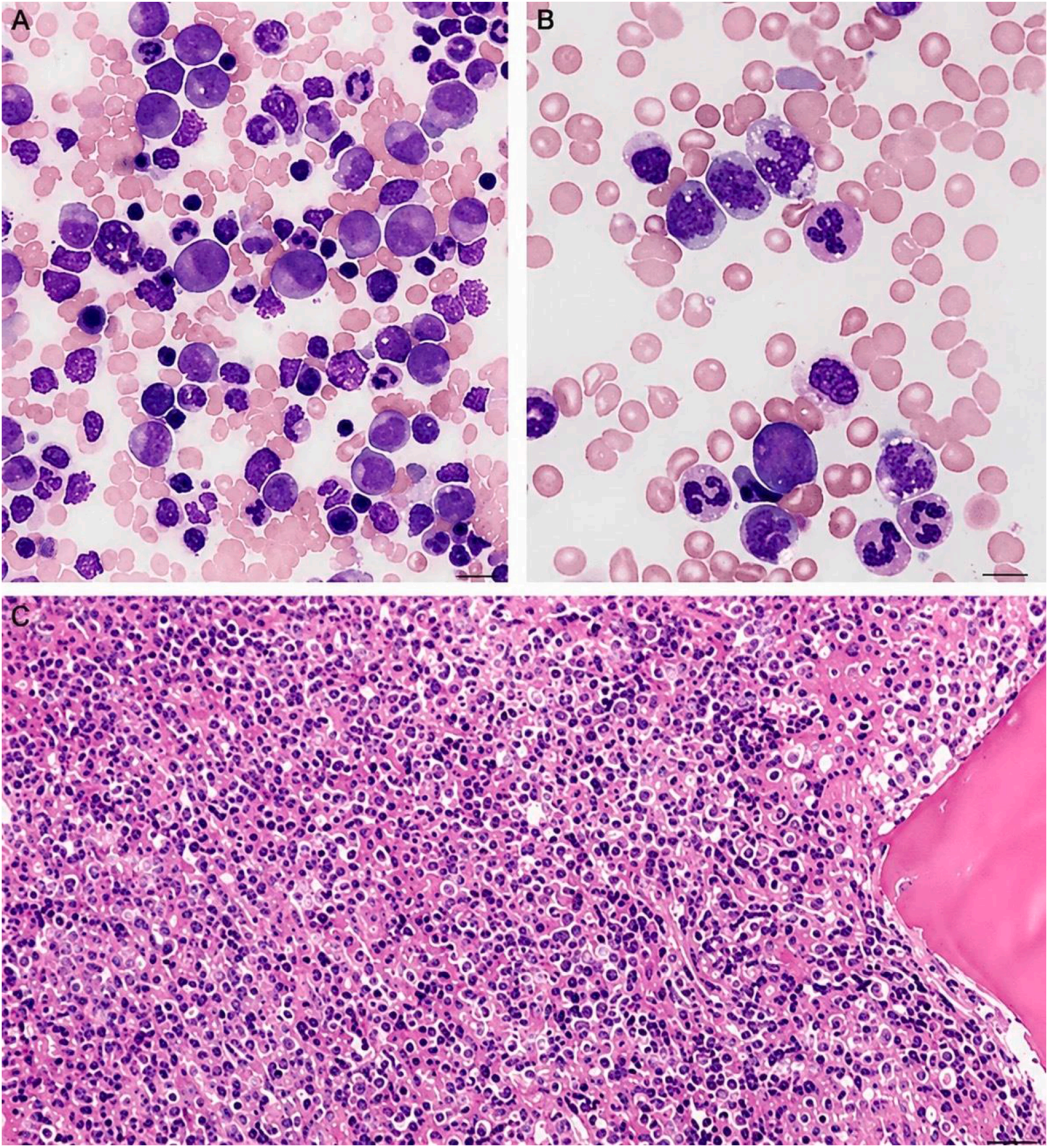
Hematopathologic findings of the patient.

Lab snapshots at intake paint the picture (Table 1): a monocyte-flooded leukocytosis, a leftward blast shift (2% in blood), and that hallmark fetal hemoglobin spike to 22% (way above the A (p.G12D) at 42% VAF. [Fig. 1]

**Table 1:** Key labs at diagnosis.

Parameter	Result	Reference range (Age-adjusted)
White blood cell count	58 × 10⁹/L	6–17 × 10⁹/L
Absolute monocyte count	12.8 × 10⁹/L	
Hemoglobin	8.2 g/dL	10.5–13.5 g/dL
Platelet count	145 × 10⁹/L	150–450 × 10⁹/L
Fetal hemoglobin (HbF)	22%	<5%
Peripheral blasts	2%	0%

A) Bone marrow aspirate showing marked monocytic proliferation with dysplastic features (Wright–Giemsa stain, × 1000). B) Peripheral blood smear demonstrating circulating immature monocytes (Wright–Giemsa, × 1000). C) Bone marrow biopsy exhibiting hypercellularity with a predominance of monocytes (H&E stain, × 400).

Next-generation sequencing identified a somatic NRAS p.G12D mutation (variant allele frequency 42%). [Figs. 2A, 2B]

**Fig. 2A:**
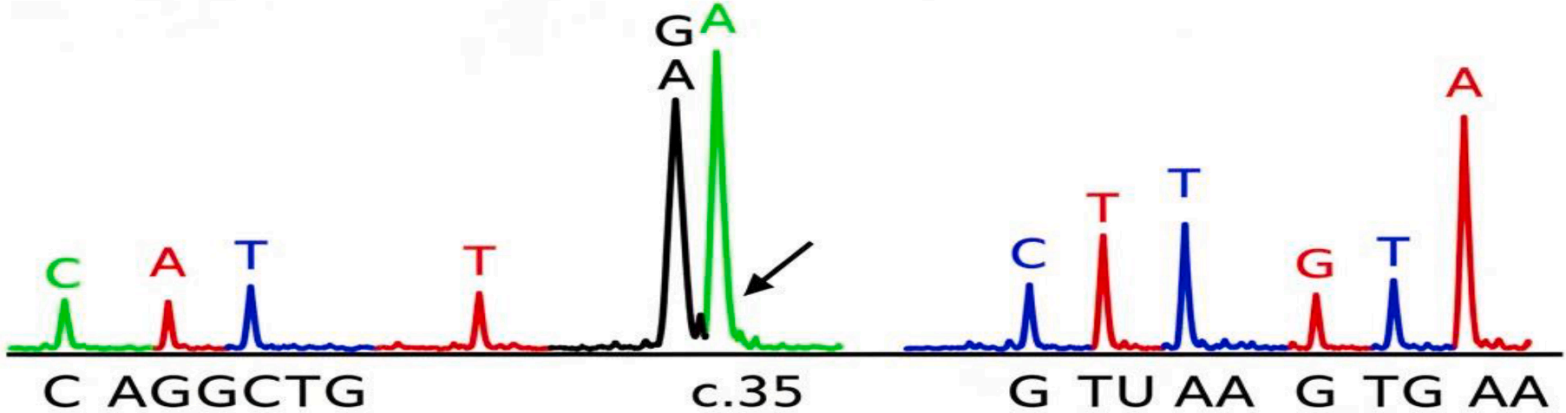
Sanger sequencing chromatogram showing the NRAS c.35G>A (p.G12D) mutation. The arrow indicates the heterozygous G>A substitution at codon 12, leading to a glycine-to-aspartic acid change.

**Fig. 2B:**
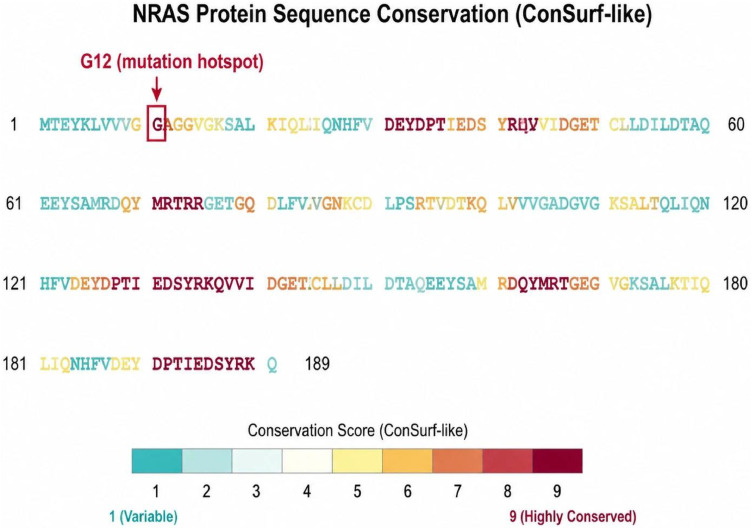
Evolutionary conservation map of the NRAS protein highlighting the G12 residue (boxed, red arrow). The Gly12 position is highly conserved across species, underscoring its pathogenic relevance. Conservation scores were generated using a ConSurf-like algoritm.

**Fig. 3:**
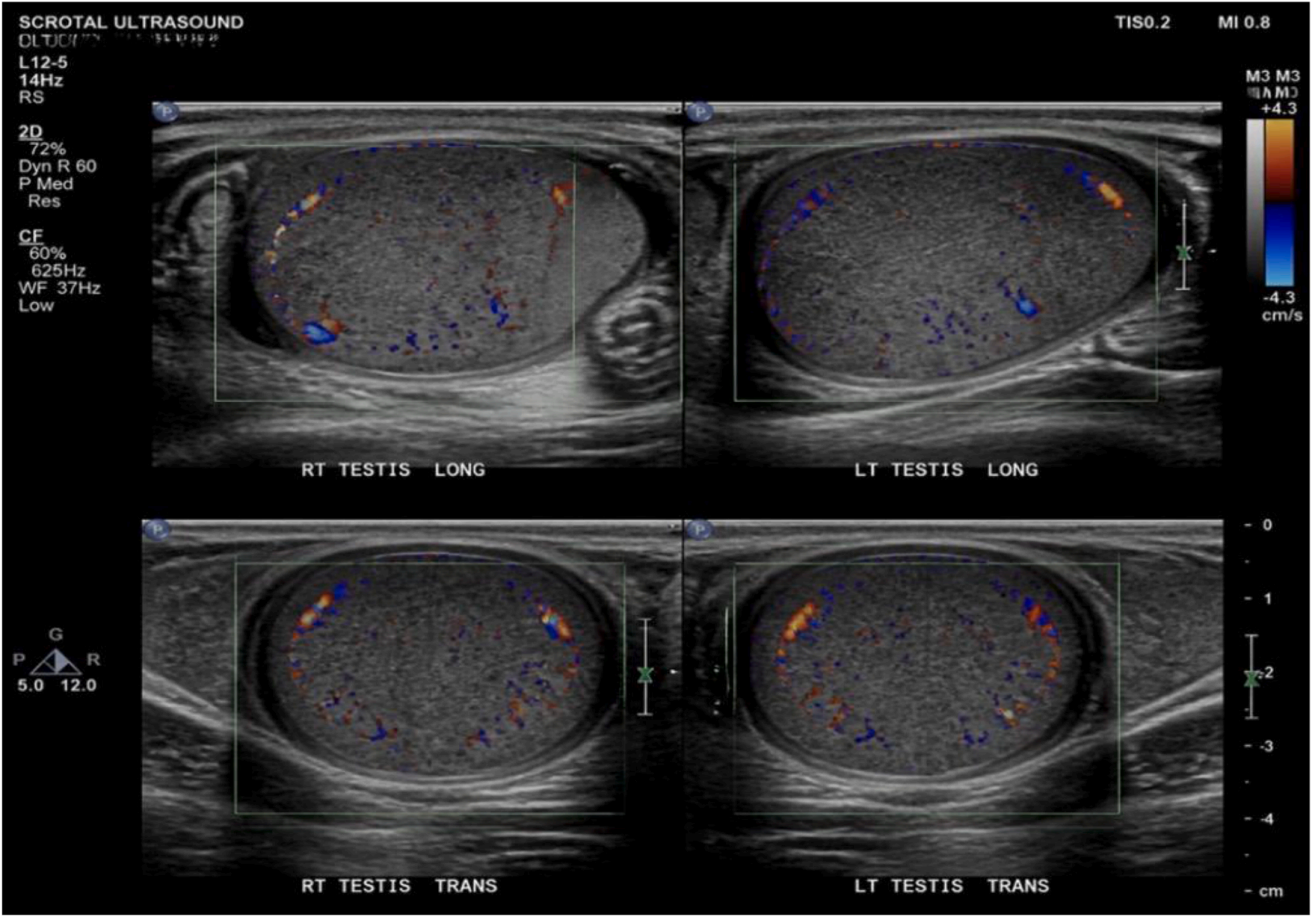
Scrotal ultrasound findings.

Scrotal ultrasound demonstrated diffuse hypoechogenicity with preserved vascularity and testicular volumes of 18.8 mL (right) and 18.2 mL (left) — approximately 10-fold above age-matched norms [11]. These characteristic imaging features were considered diagnostic of leukemic infiltration, obviating testicular biopsy in this young child. [Fig. 3]

Longitudinal and transverse color Doppler ultrasound images of both testes demonstrating diffuse bilateral testicular enlargement and homogeneous hypoechogenicity with preserved intratesticular vascularity. No focal mass lesion is identified. Findings are compatible with leukemic infiltration.

The patient received two cycles of azacitidine (75 mg/m²/day × 5 days, every 28 days), which normalized leukocyte counts, reduced splenomegaly to 2 cm, and partially decreased testicular size.

He subsequently underwent paternal haploidentical HSCT with reduced-intensity conditioning (fludarabine 150 mg/m², treosulfan 42 g/m², thiotepa 10 mg/kg × 1). GVHD prophylaxis consisted of PTCy 50 mg/kg on days +3 and +4, mycophenolate mofetil, and cyclosporine. Neutrophil and platelet engraftment occurred on days +18 and +25, respectively. Testicular volumes normalized to 1.5–1.8 mL by week 6 post-HSCT. At day +90, STR-PCR showed 100% donor chimerism and ddPCR confirmed undetectable NRAS mutation.

## 3 Discussion

This is a rare case of testicular infiltration as the presenting feature of JMML, establishing the testis as a previously unrecognized sanctuary site in this myeloid neoplasm. The rapid, radiotherapy-free resolution after HSCT underscores the potency of the graft-versus-leukemia effect even in immune-privileged niches.

The biological basis of testicular infiltration in JMML remains speculative. The testis is an immune-privileged organ protected by the blood-testis barrier, which may provide a sanctuary for leukemic cells and reduce immune-mediated clearance. Aberrant activation of the RAS/MAPK pathway, particularly through NRAS mutations, may enhance leukemic cell migration, adhesion, and tissue homing. One potential mechanism involves activation of the CXCR4/CXCL12 axis, which plays a key role in leukocyte trafficking and retention within extramedullary tissues. Similar mechanisms have been implicated in testicular involvement in acute lymphoblastic leukemia, although evidence in JMML is currently lacking. Our observation raises the possibility that dysregulated RAS signaling may facilitate migration of JMML cells into immune-privileged sites such as the testis, a hypothesis that warrants further mechanistic investigation ]^6^,^7^].

Azacitidine effectively reduced disease burden before transplant, consistent with results of the AZA-JMML-001 trial [10]. Haploidentical HSCT with PTCy provided timely curative therapy and achieved excellent early outcomes, in line with contemporary pediatric series [9]. Although the rarity of this presentation does not justify routine scrotal ultrasound in all male JMML patients, clinicians should maintain a low threshold for imaging when scrotal swelling is present or when response to therapy is unexpectedly slow. Ongoing international registries (EWOG-MDS/EBMT) will be critical to determine the true incidence and prognostic significance of this finding.

Limitation include the single-case design and short follow-up (+110 days). Longer observation is required to confirm durable remission, particularly in NRAS-mutated disease.

## 4 Conclusions

This report establishes testicular infiltration as a RARE initial manifestation of JMML and identifies the testis as a potential sanctuary site in this myeloid malignancy. Sequential azacitidine and haploidentical HSCT with PTCy achieved rapid clinical and molecular remission in this high-risk NRAS-mutant case. A low threshold for scrotal ultrasound is recommended in male patients with suggestive symptoms. Multicenter collaboration is encouraged to document atypical extramedullary sites and refine risk stratification.

## Ethics approval and consent

Written informed consent for publication was obtained from the parents. Institutional ethics approval was waived for this single-case report per local policy.

## Consent for publication

Written informed consent for publication was obtained from the patient’s parents.

## Funding

None received.

## Declaration of competing interest

The authors declare no conflicts of interest. No financial or personal relationships with other people or organizations could inappropriately influence (bias) this work. Funding for this case report was none, as stated in the manuscript. Ethics approval was not applicable, as this is a retrospective single case report with patient consent obtained for publication, and all data are anonymized in compliance with institutional and journal guidelines.

## Data Availability

All data are included in the article.
